# Hematopoietic-specific heterozygous loss of *Dnmt3a* exacerbates colitis-associated colon cancer

**DOI:** 10.1084/jem.20230011

**Published:** 2023-08-24

**Authors:** Yang Feng, Qingchen Yuan, Rachel C. Newsome, Troy Robinson, Robert L. Bowman, Ashley N. Zuniga, Kendra N. Hall, Cassandra M. Bernsten, Daniil E. Shabashvili, Kathryn I. Krajcik, Chamara Gunaratne, Zachary J. Zaroogian, Kartika Venugopal, Heidi L. Casellas Roman, Ross L. Levine, Walid K. Chatila, Rona Yaeger, Alberto Riva, Christian Jobin, Daniel Kopinke, Dorina Avram, Olga A. Guryanova

**Affiliations:** 1 Department of Pharmacology and Therapeutics, University of Florida College of Medicine, Gainesville, FL, USA; 2 Division of Gastroenterology, Hepatology, and Nutrition, Department of Medicine, University of Florida College of Medicine, Gainesville, FL, USA; 3 Department of Anatomy and Cell Biology, University of Florida College of Medicine, Gainesville, FL, USA; 4 Human Oncology and Pathogenesis Program, Memorial Sloan Kettering Cancer Center, New York, NY, USA; 5 Department of Medicine, Memorial Sloan Kettering Cancer Center, New York, NY, USA; 6 Interdisciplinary Center for Biotechnology Research, University of Florida, Gainesville, FL, USA; 7 University of Florida Health Cancer Center, Gainesville, FL, USA; 8 Immunology Department, Moffitt Cancer Center, Tampa, FL, USA

## Abstract

Clonal hematopoiesis (CH) is defined as clonal expansion of mutant hematopoietic stem cells absent diagnosis of a hematologic malignancy. Presence of CH in solid tumor patients, including colon cancer, correlates with shorter survival. We hypothesized that bone marrow–derived cells with heterozygous loss-of-function mutations of *DNMT3A*, the most common genetic alteration in CH, contribute to the pathogenesis of colon cancer. In a mouse model that combines colitis-associated colon cancer (CAC) with experimental CH driven by *Dnmt3a*^*+/Δ*^, we found higher tumor penetrance and increased tumor burden compared with controls. Histopathological analysis revealed accentuated colonic epithelium injury, dysplasia, and adenocarcinoma formation. Transcriptome profiling of colon tumors identified enrichment of gene signatures associated with carcinogenesis, including angiogenesis. Treatment with the angiogenesis inhibitor axitinib eliminated the colon tumor-promoting effect of experimental CH driven by *Dnmt3a* haploinsufficiency and rebalanced hematopoiesis. This study provides conceptually novel insights into non-tumor-cell-autonomous effects of hematopoietic alterations on colon carcinogenesis and identifies potential therapeutic strategies.

## Teaser

A preclinical mouse model demonstrates that genetic alterations in the blood system characteristic of clonal hematopoiesis (CH) contribute to an aggressive solid tumor phenotype. It further identifies cancer angiogenesis as a potential therapeutic target to mitigate adverse CH effects.

## Graphical Abstract

**Figure:**
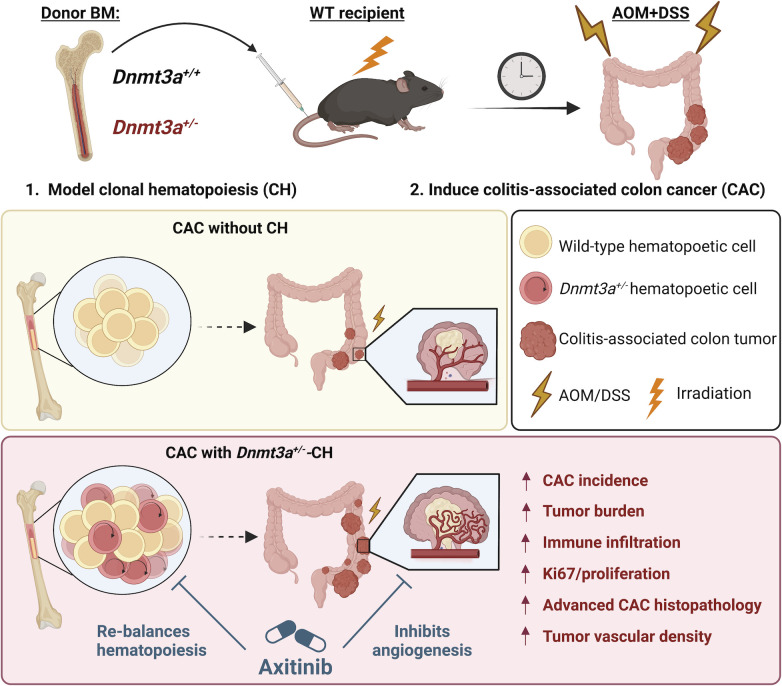

## Introduction

Clonal hematopoiesis (CH) is defined as expanded hematopoietic clone in the absence of an overt hematologic malignancy (Bowman et al., 2018; Jaiswal and Ebert, 2019; Köhnke and Majeti, 2021; Steensma et al., 2015). With ∼10^5^ hematopoietic stem cells (HSCs) in adult bone marrow (BM), an estimated one million protein-coding mutations are acquired by the stem cell pool by the age of 60 (Lee-Six et al., 2018; Watson et al., 2020; Xu et al., 2018). While most mutations are functionally neutral or occasionally detrimental, some genetic alterations may confer fitness advantage, allowing select HSCs to outcompete their peers and dominantly contribute to the pool of mature blood cells in the periphery (Ahmad et al., 2023; Fabre et al., 2022; McKerrell et al., 2015; Mitchell et al., 2022; Robertson et al., 2022; Watson et al., 2020). CH is most frequent in the elderly (10–40%) and is commonly driven by somatic mutations in leukemia-associated genes such as *DNMT3A* (Bick et al., 2020; Busque et al., 1996, 2012; Coffee et al., 2017; Genovese et al., 2014; Guermouche et al., 2020; Jaiswal et al., 2014; McKerrell et al., 2015; van Zeventer et al., 2021; Xie et al., 2014; Young et al., 2016; Zink et al., 2017).

While it seems intuitive that CH is associated with an increased risk of leukemia (Abelson et al., 2018; Corces-Zimmerman et al., 2014; Desai et al., 2018; Genovese et al., 2014; Gillis et al., 2017; Jan et al., 2012; Shlush et al., 2014; Takahashi et al., 2017; Wong et al., 2018; Xie et al., 2014), growing evidence shows that it is also linked to multiple disease conditions outside the hematopoietic system and increased overall mortality (Agrawal et al., 2022; Dawoud et al., 2022; Jaiswal et al., 2014, 2017; Kim et al., 2021; Miller et al., 2022). This includes cardiovascular disease (Dorsheimer et al., 2019; Jaiswal et al., 2017; Mas-Peiro et al., 2020; Yokokawa et al., 2021), infections (Bolton et al., 2021), ulcerative colitis (UC; Zhang et al., 2019), and a wide spectrum of solid tumors (Comen et al., 2020; Coombs et al., 2017, 2018; Kleppe et al., 2015; Ptashkin et al., 2018; Severson et al., 2018; Swisher et al., 2016; Yokokawa et al., 2021; Zajkowicz et al., 2015). In patients with solid tumors, the prevalence of CH similarly increases with age yet is notably more frequent (∼30% of cases) and correlates with shorter survival due primarily to solid tumor progression (Bolton et al., 2019; Bowman et al., 2018; Coombs et al., 2017), raising the clinically and biologically important question if these two conditions are causally linked. The effect is the strongest for CH with presumed leukemic driver mutations, such as in the epigenetic modifier gene *DNMT3A*, and increases with CH clone size conventionally quantified by calculating variant allele frequency (Bowman et al., 2018; Jaiswal and Ebert, 2019; Köhnke and Majeti, 2021; Steensma et al., 2015).

*DNMT3A*, a de novo DNA methyltransferase that epigenetically enforces hematopoietic stem cell differentiation programs (Challen et al., 2011; Guryanova et al., 2016a; Hormaechea-Agulla et al., 2021; Jeong et al., 2018; Ketkar et al., 2020; Leoni et al., 2017; Lim et al., 2021), is recurrently mutated in hematologic malignancies (Brunetti et al., 2017; Cancer Genome Atlas Research, 2013; Ley et al., 2010; Ribeiro et al., 2012; Spencer et al., 2017; Venugopal et al., 2021; Walter et al., 2011; Yan et al., 2011). *DNMT3A* is by far the most frequently altered gene in CH (Buscarlet et al., 2017; Coombs et al., 2017; Genovese et al., 2014; Jaiswal et al., 2014; van Zeventer et al., 2021; Xie et al., 2014; Young et al., 2016) with the majority of mutations consistent with a heterozygous loss of function (∼50%, truncating indels, splice, and nonsense; Brunetti et al., 2017; Venugopal et al., 2021). Yet, despite important clinical implications, the causal relationship between presence of CH and aggressive phenotype of unrelated solid tumors has not been rigorously addressed.

Colon cancer is one of the leading causes of cancer-related deaths in developed countries (Ahmed, 2020; Keum and Giovannucci, 2019; Siegel et al., 2019). Inflammatory bowel disease (IBD), including UC and Crohn’s disease, is a well-known risk factor for colon cancer (Beaugerie and Itzkowitz, 2015). Colitis-associated colon cancer (CAC) accounts for 15% of overall mortality among all IBD patients. Compared with sporadic colorectal cancer, patients with CAC often present with multifocal tumors arising from precancerous lesions that are challenging to detect and remove endoscopically, and tend to rapidly develop chemoresistance (Yaeger et al., 2020). Over 20% of patients with colon cancer in the publicly available Memorial Sloan Kettering Cancer Center (MSKCC) clinical sequencing database have detectable CH (Cerami et al., 2012; Gao et al., 2013), which is notably more prevalent than in age-matched cancer-free population. Despite screening and lifestyle interventions, most patients present with advanced disease associated with poor outcome. To inform the choice of optimal therapeutic approaches and improve survival rates, better understanding of disease-modifying factors that contribute to the aggressive tumor phenotype is critically needed.

Potential involvement of CH in the pathogenesis of coincident solid tumors has a far-reaching translational impact, yet a better understanding of this relationship is hindered by the lack of animal models. Here, we combined a well-established induction of CAC (De Robertis et al., 2011; McIntyre et al., 2015; Parang et al., 2016) with a BM transplantation (BMT) approach to experimental CH driven by heterozygous *Dnmt3a* loss (Guryanova et al., 2016a, 2016b). This unique tool enables rigorous interrogation into the role of CH in the pathogenesis of solid tumors driven by unrelated genetic alterations, uncoupled from environmental, lifestyle, iatrogenic, or other confounding factors (Bolton et al., 2020; Coombs et al., 2017; Dawoud et al., 2020; Gillis et al., 2017; Hong et al., 2022; Hormaechea-Agulla et al., 2021; Hsu et al., 2018; King et al., 2020). At the same time, genetic alterations in BM-derived cells are likely to affect their function and contribute to disease conditions outside of the hematopoietic system. While attempts to unveil the role of CH in non-malignant conditions such as atherosclerosis or gout were made (Agrawal et al., 2022; Fuster et al., 2017; Jaiswal et al., 2017; Rauch et al., 2018; Sano et al., 2018), similar studies in cancer models are limited. In this proof-of-concept study, we report that heterozygous inactivation of *Dnmt3a* restricted to the hematopoietic compartment exacerbates the development of colon cancer phenotype in an inflammation-induced mouse model. We further credential an anti-angiogenesis drug axitinib as a potential therapeutic mitigation strategy.

## Results and discussion

### Heterozygous loss of *Dnmt3a* in the BM leads to accentuated CAC phenotype

**Figure 1.**
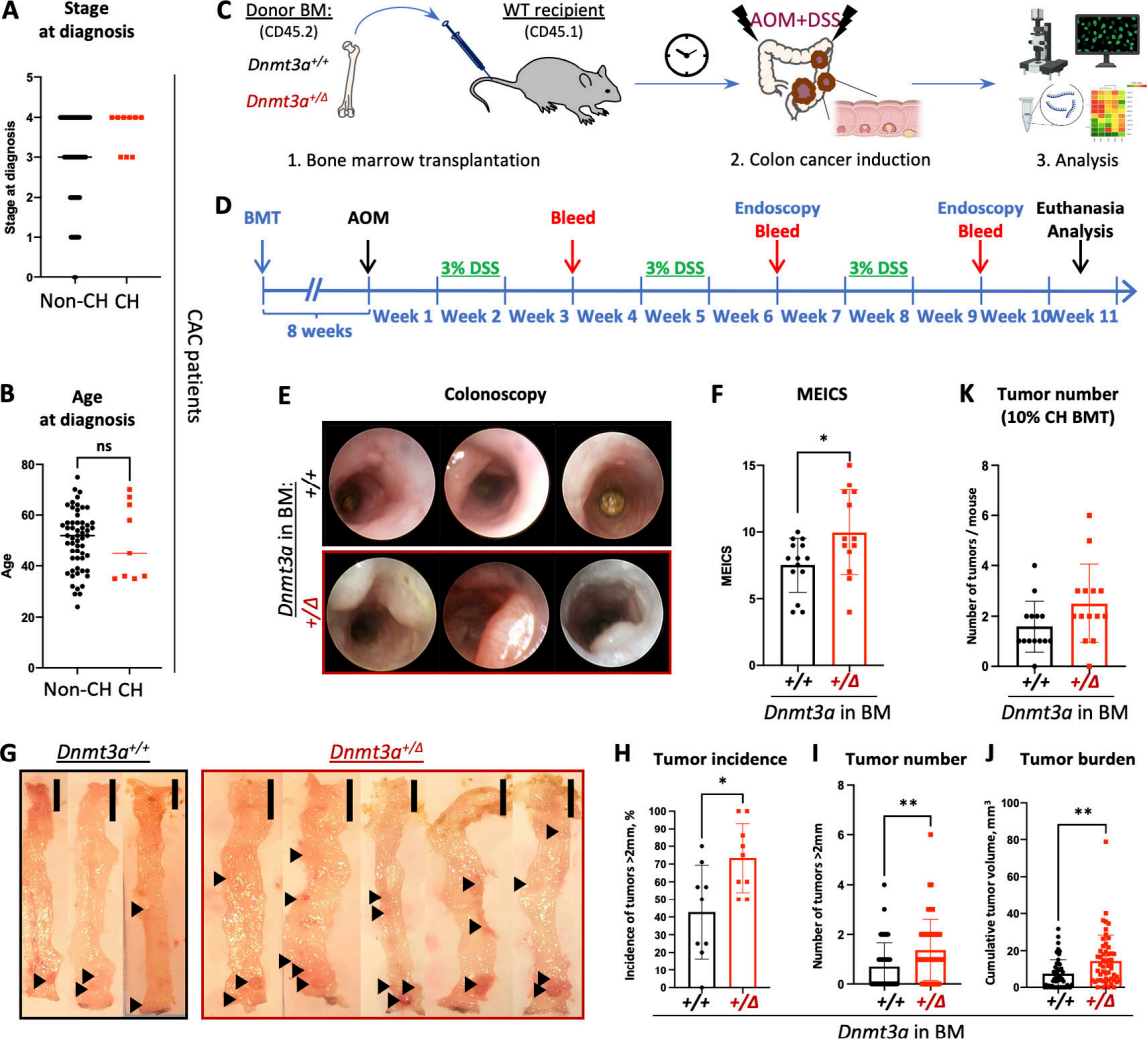
***Dnmt3a* haploinsufficiency specifically in the blood system promotes tumorigenesis in a model of CAC. (A)** CAC patients with detectable CH at diagnosis (*n* = 9) tend to be more likely to present with an advanced stage of the disease (stage 3 or 4) than those without CH (*n* = 57; Mann–Whitney test, P = 0.074). **(B)** Age (yr) of CAC patients with or without CH at diagnosis (*n* = 57 or 9, Mann-Whitney test, P = 0.9). **(C and D)** Experimental workflow using a mouse model of BMT-based CH and AOM/DSS-induced CAC (C) and timeline (D). **(E and F)** Representative colonoscopy findings by the Tele Pack Vet X mini-endoscopic system in a mouse model of CH (E) and corresponding MEICS scores in *Dnmt3a*^*+/+*^ and *Dnmt3a*^*+/∆*^ BM chimeras (F, *n* = 14–13 from two replicate experiments, Student’s *t* test, P = 0.05). The diameter of the endoscopy probe is 1.9 mm. **(G–J)** Representative gross pathology of colons with tumors (black arrowheads, G, bar, 10 mm), proportion of animals presenting with tumors (>2 mm in diameter) in nine replicate experiments (H, Mann–Whitney test, P = 0.019), number of large tumors (>2 mm in diameter) per animal (I, *n* = 55–53, Mann–Whitney test, P = 0.0015), and cumulative tumor burden per animal (J, *n* = 55–53, Mann–Whitney test, P = 0.0017). **(K)** Mice transplanted with a mixture of 10% *Dnmt3a*^*+/Δ*^ and 90% WT competitor BM (10% CH BMT) develop more tumors following CAC induction by AOM/DSS, compared with 10% *Dnmt3a*^*+/+*^ controls (*n* = 14/group in one experiment, Mann–Whitney test, P = 0.058). *, P ≤ 0.05; **, P ≤ 0.01; ns, not significant.

To explore the relationship between genetic alterations in the blood system and the severity of coincident CAC, we examined presence of CH mutations in paired blood and tumor samples in a cohort of 66 patients with CAC treated at MSKCC. We observed a strong trend toward a more advanced disease at diagnosis among patients with detectable CH by MSKCC-IMPACT (Cerami et al., 2012; Gao et al., 2013; Fig. 1 A, P = 0.056, one-sided Fisher’s exact test comparing patients with local [stages 0, 1, and 2] and advanced [stages 3 and 4] disease, and Table 1). Patient age at diagnosis could not explain the difference in CH prevalence (Fig. 1 B), consistent with previous reports implicating that the impact of CH on solid tumor outcomes was not due to differences in age. There was no correlation between the size of CH clone measured as the variant allele frequency and CAC stage (P = 0.51, Mann–Whitney test) or prior chemotherapy exposure (P = 0.27, Mann–Whitney test), bearing in mind that the cohort was too small to lend sufficient statistical power for such analyses, and CH mutations were only seen in patients with advanced stage disease (3 and 4). To directly test the causal relationship between presence of CH and CAC pathogenesis, we performed a proof-of-concept animal study.

**Table 1.** Clinical characteristics and presence of CH in a cohort of 66 patients with CAC

CAC case #	Gene	Protein change	Variant allele frequency	IBD type	Age at cancer diagnosis (years)	Cancer stage at diagnosis	Primary site	Classification of primary site	Vital status (0 = alive, 1 = dead)	Overall survival from cancer diagnosis (months)	Site of first metastasis	Chemo exposure before sequencing
1				UC	55	3	R colon	Right (R) side	1	30.4	Liver	Y
2				UC	55	3	Rectum	Left (L) side	1	43.7	Bone	Y
3				UC	44	1	Rectum	L side	1	116.4	Lung	N
4				UC	29	3	Rectum	L side	0	155.7	Pelvis	Y
5				UC	48	3	Sigmoid colon	L side	1	35.5	Peritoneum, small intestine	Y
6				Crohn’s	57	2	Rectum	L side	0	61.1	N/A	N
7				Crohn’s	55	3	Ascending colon	R side	1	18.7	Lung, peritoneum	Y
8	MGA	R2529Kfs*9	0.124	Crohn’s	64	4	Descending colon	L side	1	1.9	Peritoneum	N
9	CHEK2	A392V	0.071	Crohn’s	70	4	Sigmoid	L side	0	120.3	Liver	Y
10	DNMT3A	C494F	0.035	UC	36	4	Rectosigmoid	L side	1	131.2	Liver	Y
11				Crohn’s	48	4	L colon	L side	1	18.1	Peritoneum	N
12				Crohn’s	29	3	Sigmoid	L side	1	34.0	Peritoneum, pelvis	Y
13				Crohn’s	37	2	Ileum	R side	0	0.7	N/A	N
14				UC	31	4	Transverse colon	R side	1	32.2	Liver	Y
15				Crohn’s	54	1	Rectal	L side	0	67.0	N/A	N
16				Crohn’s	43	3	Rectal	L side	1	55.0	Lymph nodes	Y
17	MDC1	R1882*	0.02	Crohn’s	35	4	Rectal	L side	1	19.9	Liver, lung, bone	N
18				UC	55	4	Sigmoid	L side	1	5.0	Pelvis, ovary	Y
19	ABL1	S1120L	0.043	Crohn’s	58	3	Rectal	L side	1	31.0	Pelvis	Y
20				Crohn’s	57	3	Rectal	L side	1	28.3	Peritoneum	N
21				Crohn’s	57	3	Small intestine	Small intestine	1	56.8	Peritoneum	Y
22				Crohn’s	52	2	Ileum	Small intestine	0	52.8	N/A	N
23				Crohn’s	38	2	Ileum	Small intestine	0	50.8	N/A	N
24				UC	45	1	Rectal	L side	0	60.3	N/A	N
25				Crohn’s	57	4	Ileum	Small intestine	1	17.5	Liver	N
26	DNMT3A	R742Gfs*37	0.03	Crohn’s	35	4	Rectum	L side	1	13.5	Liver	Y
27				Crohn’s	38	3	Rectal	L side	0	83.9	Pelvis	Y
28	DNMT3B	R497Q	0.028	UC	67	3	Rectosigmoid	L side	0	43.4	N/A	N
29				Crohn’s	51	3	Rectum	L side	0	40.7	Lymph nodes	N
30				UC	51	3	Sigmoid	L side	1	34.5	Liver	Y
31				UC	65	4	Distal transverse	L side	1	44.9	Liver	Y
32				UC	52	3	Rectum	L side	1	20.9	Lung, liver, bone	N
33				Crohn’s	64	2	Small intestine	Small intestine	1	18.6	Bone, liver, lymph nodes, lungs	N
34				Crohn’s	43	4	Rectum	L side	0	23.2	Skin	Y
35				Crohn’s	46	4	Transverse colon	R side	0	32.4	Peritoneum	N
36				UC	62	4	Rectum	L side	1	42.9	Liver, lymph nodes	Y
37				Crohn’s	70	2	Ileum	Small intestine	0	88.6	Lung, lymph nodes	Y
38				UC	75	4	Sigmoid	L side	1	8.5	Pelvis	Y
39				Crohn’s	57	3	Anal fistula	Anal fistula	0	33.3	Lymph nodes	Y
40				UC	63	0	Descending colon	L side	0	25.0	N/A	N
41				Crohn’s	60	4	Ileum	Small intestine	1	13.4	Liver	Y
42				UC	67	1	Rectosigmoid	L side	0	60.8	N/A	N
43				UC	49	1	Rectum	L side	0	57.4	N/A	N
44				UC	56	3	Transverse colon, descending	L side	0	20.5	N/A	N
45				Crohn’s	37	2	Cecum	R side	0	21.5	N/A	N
46				Crohn’s	39	4	Cecum	R side	0	9.7	Liver	Y
47				UC	36	4	Ascending colon	R side	1	7.2	Liver	Y
48				UC	63	2	Descending colon	L side	0	22.7	N/A	Y
49				UC	54	4	Rectum	L side	0	19.5	Liver	N
50				Crohn’s	53	4	Splenic flexure	L side	0	26.4	Peritoneum	Y
51				Crohn’s	38	3	Sigmoid	L side	0	1.6	N/A	N
52				UC	52	4	Rectum	L side	0	19.3	Lymph nodes	N
53				UC	32	4	Transverse colon	R side	0	22.2	Liver	N
54	CREBBP	R2344W	0.013	Crohn’s	36	3	Ascending colon	R side	0	2.8	N/A	N
	SH2B3	H158P	0.023									
55				UC	50	3	Rectum	L side	0	18.2	N/A	N
56				Crohn’s	43	4	Cecum	R side	0	35.3	Liver	Y
57				UC	56	4	Sigmoid	L side	0	19.1	Peritoneum, ovaries	N
58	DNMT3A	R882H	0.015	UC	45	4	Rectum	L side	0	28.9	Lymph nodes	Y
	PBRM1	R836Q	0.076									
59				UC	36	4	Sigmoid	L side	1	12.2	Peritoneum	N
60				Crohn’s	64	1	Rectum	L side	0	20.3	N/A	N
61				Crohn’s	49	1	Ileum	Small intestine	0	5.3	N/A	N
62				Crohn’s	63	4	Cecum / terminal ileum	R side	0	5.9	Liver, regional nodal, peritoneum	N
63				UC	69	CIS (0)	Rectum	L side	0	2.7	N/A	N
64				UC	46	4	Rectosigmoid	L side	0	3.7	Peritoneum	N
65				UC	32	4	Ascending colon	R side	1	14.8	Peritoneum	N
66				Crohn’s	24	4	Hepatic flexure	R side	0	0.5	Lymph nodes	N

**Figure S1.**
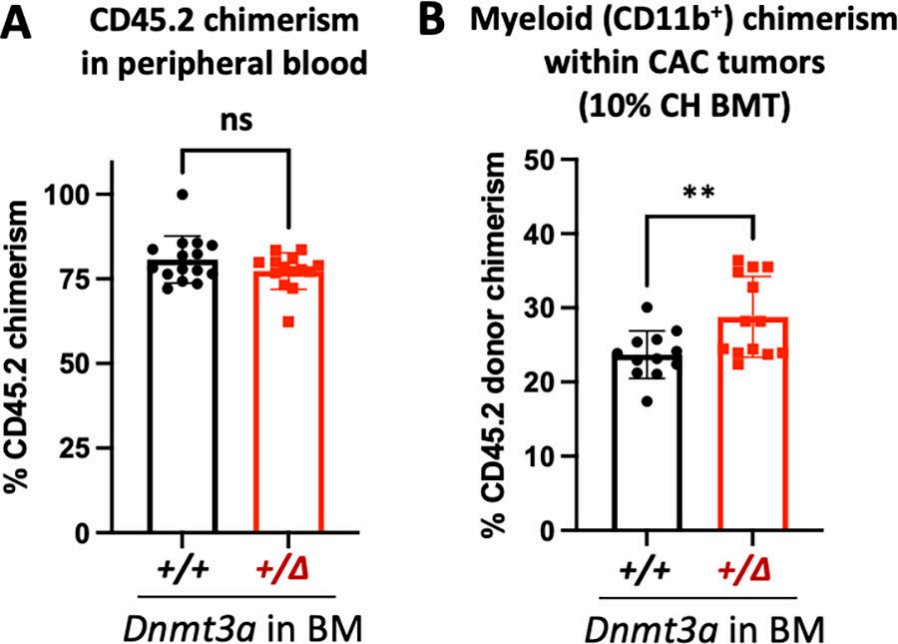
***Dnmt3a*-CH–derived myeloid cells preferentially traffic to colon tumors. (A)** Donor chimerism in the peripheral blood of tumor-bearing mice transplanted with *Dnmt3a*^*+/Δ*^ and control *Dnmt3a*^*+/+*^ BM (CD45.2) into WT recipients (CD45.1) (*n* = 15–14, one experiment, Mann–Whitney test, P = 0.35). **(B)** *Dnmt3a*-CH-derived myeloid cells (CD11b^+^) exhibit enhanced tumor tropism in the 10% CH BMT CAC model, as seen by increased CD45.2 chimerism (*n* = 12–13, one experiment, two-tailed *t* test with Welch’s correction, P = 0.009). **, P ≤ 0.01; ns, not significant.

To experimentally model CH in mice, we adopted a BMT-based approach using *Dnmt3a*^*+/f*^*:Mx1-Cre*^*+*^ and *Dnmt3a*^*+/+*^*:Mx1-Cre*^*+*^ (WT control) mice as donors after successful Cre-recombination induced by intraperitoneal (IP) administration of poly(I:C), yielding *Dnmt3a*^*+/Δ*^ and *Dnmt3a*^*+/+*^ mice. Fully engrafted *Dnmt3a*^*+/Δ*^ and control *Dnmt3a*^*+/+*^ BM chimeras (Fig. S1 A) underwent a CAC induction protocol initiated by a single injection of azoxymethane (AOM, 10 mg/kg, IP) followed by three cycles of dextran sulfate sodium (DSS) salt exposure (3% in sterile drinking water; Santiago et al., 2019; Tanaka et al., 2003; Fig. 1, C and D). AOM/DSS CAC is a gold-standard autochthonous model with high penetrance and predictable latency (De Robertis et al., 2011; McIntyre et al., 2015; Parang et al., 2016). The use of immunocompetent animals preserves all modes of tumor-microenvironment interactions. AOM/DSS chemically induced CAC produces a spectrum of mutations that captures genetic landscapes observed in human colon cancer (De Robertis et al., 2011) and recapitulates human pathology. This unique tool enables rigorous interrogation into CH involvement in the pathogenesis of solid tumors driven by unrelated genetic alterations, which can be readily extended to other cancer types. Colonoscopy at 9 wk after initiation of the AOM/DSS treatment demonstrated increased colon wall opacity, visible bleeding, and numerous fibrin patches, indicating heightened colon pathology, and detected more, larger tumors in accessible colon regions in *Dnmt3a*^*+/Δ*^ BM chimeras compared with *Dnmt3a*^*+/+*^-engrafted controls (Fig. 1 E). Modified murine endoscopic index of CAC severity (MEICS; Fung and Putoczki, 2018) independently scored by two blinded investigators was significantly elevated in the *Dnmt3a*^*+/Δ*^ group (Fig. 1 F).

To further investigate the effect of *Dnmt3a* heterozygous loss in the hematopoietic system characteristic of CH on CAC induction, mice were euthanized for comprehensive colon evaluation 10 wk after initiation of the AOM and DSS treatment. Colon gross pathology (Fig. 1 G) demonstrated significantly elevated penetrance of the tumor phenotype (incidence of large tumors >2 mm in diameter, Fig. 1 H), higher large tumor number (Fig. 1 I), and increased total tumor burden per colon (Fig. 1 J) in *Dnmt3a*^*+/Δ*^ chimeras. Together, these findings suggest that hematopoietic-specific *Dnmt3a* haploinsufficiency promotes both cancer initiation and progression in the context of CAC. To more closely model human CH where mutant clones contribute a fraction of mature cells in the peripheral blood, we next transplanted WT recipient animals with 10% *Dnmt3a*^*+/Δ*^ (or *Dnmt3a*^*+/+*^ control) mixed with 90% WT competitor BM cells distinguishable by CD45.2 and CD45.1 pan leukocytic markers. Following the AOM/DSS CAC induction protocol, we observed a higher number of colon tumors in the *Dnmt3a*^*+/Δ*^-CH 10% BM chimeras (Fig. 1 K) and a higher tumor tropism of CH-derived myeloid cells (Fig. S1 B), demonstrating that a small *Dnmt3a*-CH clone was sufficient to promote cancer initiation.

### *Dnmt3a* haploinsufficiency in the BM leads to accentuated pathological features of CAC

**Figure 2.**
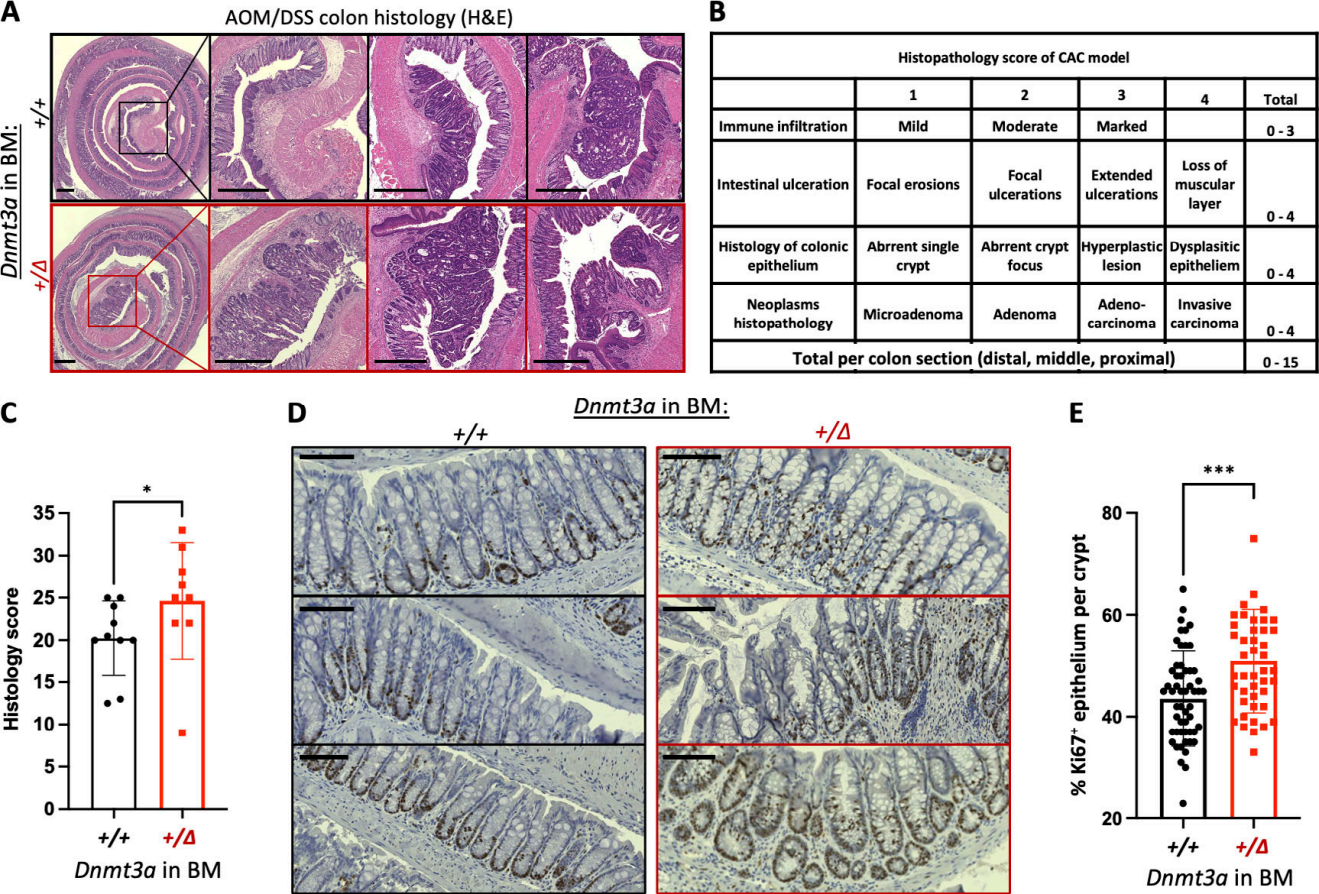
**Histopathological features of a more aggressive CAC in animals with hematopoietic-specific heterozygous *Dnmt3a* loss. (A)** Representative images of H&E-stained Swiss-rolled colons. Colons from animals engrafted with *Dnmt3a*^*+/Δ*^ BM exhibit marked immune cell infiltration, dysplastic epithelium, extensive ulceration, global hyperplasia, and occasional invasive adenocarcinoma. Bar, 500 μm. **(B)** Histopathology scoring criteria. **(C)** Overall histology scores indicate more advanced colon and tumor pathology in *Dnmt3a*^*+/Δ*^ BM-chimeras compared with WT-grafted controls (*n* = 10–9 from three replicate experiments, Mann–Whitney test, P = 0.031). **(D and E)** Representative examples of Ki67 immunohistochemistry staining in intestinal crypts after one cycle of DSS treatment (D; bar, 100 μm) and increased proliferation of colonic epithelium in animals with *Dnmt3a*^*+/Δ*^ BM (right panels), quantified as percentage of Ki67^+^ epithelial cells per crypt (E, *n* = 55–44, one experiment, Mann–Whitney test, P = 0.0005). *, P ≤ 0.05; ***, P ≤ 0.001.

Given that full *Dnmt3a*^*+/Δ*^ BM chimeras have higher tumor burden and larger tumor size compared to WT controls, we performed histopathology analysis on colons from both groups based on a modified quantitative scoring system for DSS-induced murine CAC (Arthur et al., 2012; Cooper et al., 1993; Dieleman et al., 1998; Karrasch et al., 2007). H&E-stained paraffin sections of Swiss-rolled colons showed marked immune infiltration, extensive ulceration and dysplasia of colonic epithelium, and more frequent adenocarcinoma formation with occasional submucosal invasion in *Dnmt3a*^*+/Δ*^-reconstituted animals (Fig. 2 A). In comparison, WT control chimeras showed moderate dysplasia, fewer adenoma polyps, mild ulceration, and more tissue regeneration. Histology scores based on four parameters (immune infiltration [0–3], ulceration [0–3], morphology of colonic epithelium [0–4], and neoplasms [0–4]), independently assigned by two blinded investigators, were significantly higher in the *Dnmt3a*^*+/Δ*^ group than in WT (Fig. 2, B and C). We found that in *Dnmt3a*^*+/Δ*^ BM chimeras, increased proliferation of colonic epithelium occurred early in the carcinogenesis process, evidenced by a higher proportion of Ki67-marked cells per crypt in the mucosal layer after the first cycle of DSS (Fig. 2, D and E). Overall, *Dnmt3a* haploinsufficiency in the BM yields more advanced CAC histopathology, consistent with a more severe tumor burden.

### Gene expression profiling identifies signatures of accentuated CAC tumorigenesis in animals with experimental CH driven by *Dnmt3a*^*+/Δ*^

**Figure 3.**
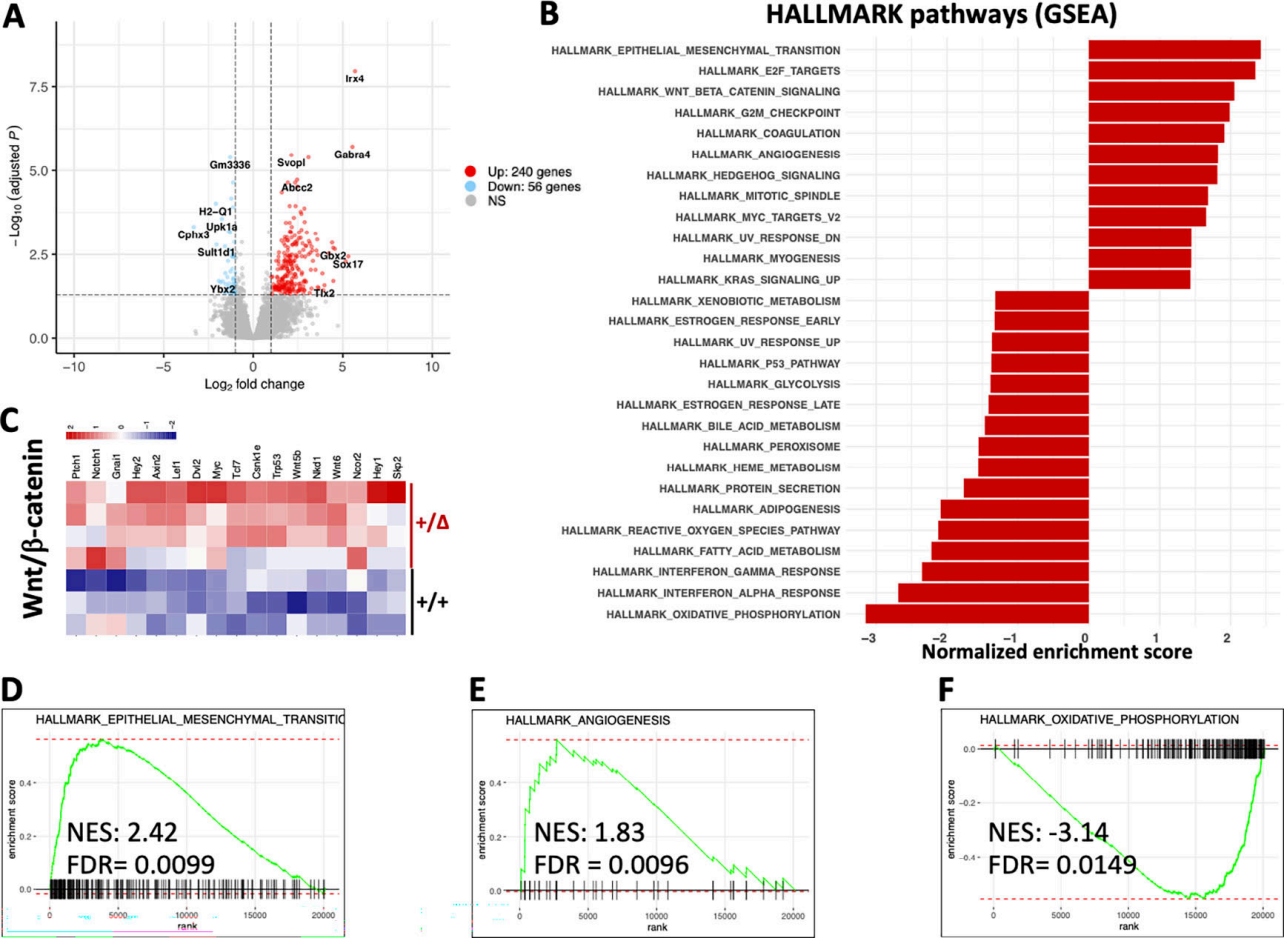
**Transcriptomic analysis identifies enrichment of gene expression signatures associated with accentuated colon tumorigenesis, angiogenesis, and changes in metabolism. (A)** Differential gene expression in colon tumors from animals with and without experimental *Dnmt3a*^*+/Δ*^ CH. Volcano plot showing 297 significantly (|log_2_ fold change| > 1, adjusted P < 0.05) up- (241) or downregulated (56) genes in *Dnmt3a*^*+/Δ*^ chimeras (*n* = 4) compared with WT-transplanted control tumors (*n* = 3). **(B)** Most significantly enriched gene sets from the HALLMARK collection (MSigDB) in tumor transcriptomes from *Dnmt3a*^*+/Δ*^ chimeras compared with control *Dnmt3a*^*WT*^-engrafted mice, |normalized enrichment score| > 1.3. **(C)** Column-normalized heat map of differentially expressed genes showing activation of the Wnt/β-catenin pathway in colon tumors from mice with *Dnmt3a*^*+/Δ*^ BM. **(D–F)** GSEA plots showing positive enrichment of the epithelial–mesenchymal transition (D) and angiogenesis (E) gene signatures and negative enrichment of the oxidative phosphorylation pathway (F). NES, normalized enrichment score; FDR, false discovery rate.

**Figure S2.**
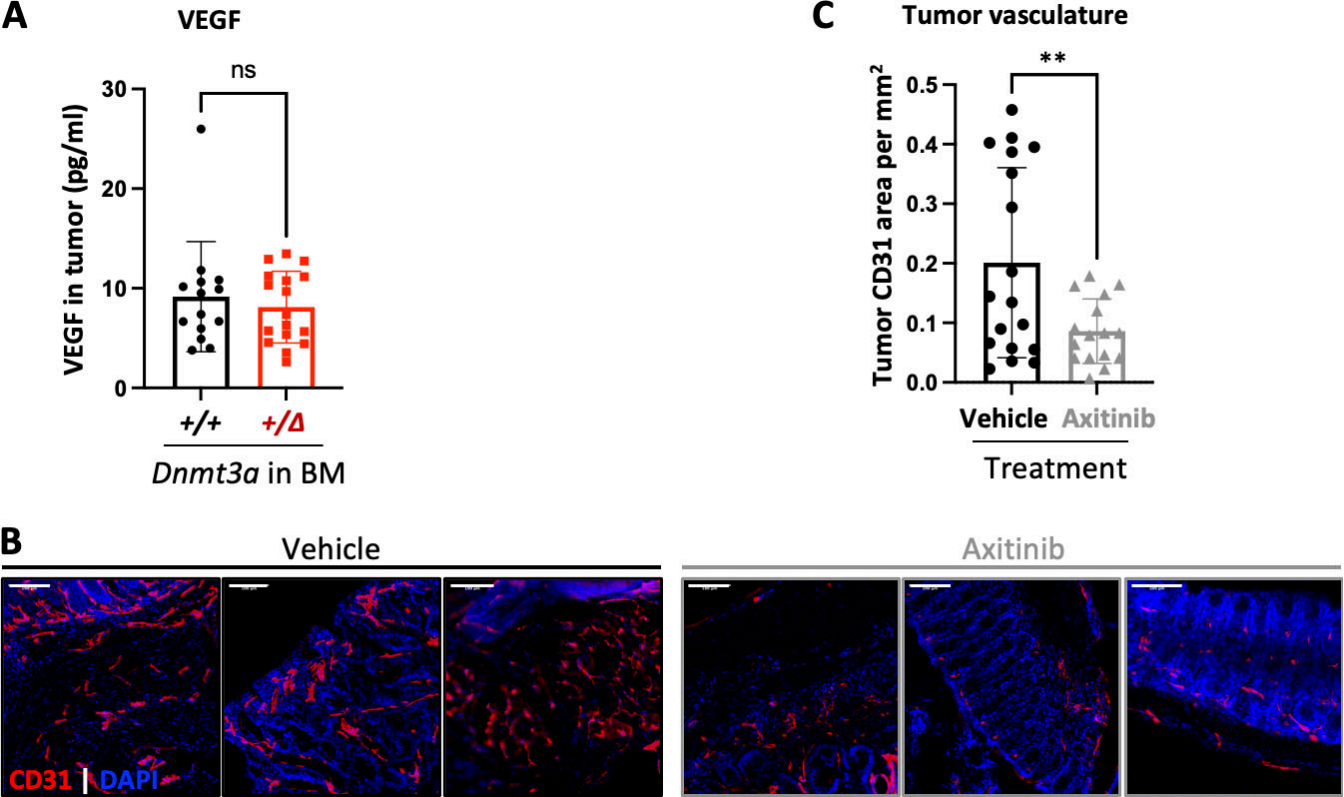
**Axitinib suppresses tumor vascularization. (A)** Intratumoral concentration of Vegf did not differ between mice transplanted with *Dnmt3a*^*+/Δ*^ and control *Dnmt3a*^*+/+*^ BM (*n* = 14–17 from two independent experiments, Student’s *t* test with Welch’s correction, P = 0.55). Vegf concentrations were determined in tumor protein lysates by multiplex ELISA performed by Eve Technology Corp. **(B and C)** Administration of a small molecule VEGFR and multikinase inhibitor axitinib potently decreases tumor angiogenesis. Mice transplanted with *Dnmt3a*^*+/+*^ BM were treated with axitinib (25 mg/kg per os, three weekly doses) throughout CAC induction. Representative images of colon tumors (B, vasculature detected by endothelial marker CD31 in red; DAPI was used to visualize nuclei in blue; bar, 100 μm). Vascular density (as CD31 staining area per mm^2^) was quantified at endpoint in 16–18 colon tumors in vehicle and treatment groups from one experiment (C, Student’s *t* test with Welch’s correction, P = 0.009). The images of colon tumors from *Dnmt3a*^*+/+*^ chimeras treated with axitinib are also presented in Fig. 4 D. **, P ≤ 0.01; ns, not significant.

To identify specific molecular mechanisms likely driving accentuated CAC tumorigenesis in animals with *Dnmt3a*^*+/Δ*^ hematopoiesis, we profiled tumor transcriptomes from *WT* and *Dnmt3a*^*+/Δ*^ chimeras by bulk RNA sequencing (RNA-seq; Table S1). We identified 297 differentially expressed genes (log_2_ fold change > 1, P-adjusted < 0.05, Fig. 3 A) with most being upregulated rather than downregulated in the *Dnmt3a*^*+/Δ*^ group (241 up and 56 down, Table S2). Gene set enrichment analysis (GSEA; Subramanian et al., 2005) using the HALLMARK collection of gene signatures (Liberzon et al., 2015; Fig. 3 B) detected significant positive enrichment of cancer- and proliferation-related pathways such as epithelial–mesenchymal transition, Wnt/β-catenin, and angiogenesis (Fig. 3, C–E), along with E2F, mitotic spindle, G2/M checkpoint, and MYC signaling, and shifts in metabolism such as negative enrichment of oxidative phosphorylation (Fig. 3 F) and adipogenesis/fatty acid metabolism (Table S3). Elevated expression of the Wnt/β-catenin pathway genes is notable (Fig. 3 C) given its known role in driving colon cancer and association with poor prognosis (Jackstadt et al., 2020), which most likely reflects a more advanced stage of the disease evidenced by increased tumor size and overall cancer burden in *Dnmt3a*^*+/Δ*^-transplanted animals (Fig. 1, G and J). The leading edge of the angiogenesis signature was dominated by vascular endothelial-specific genes along with fibroblast growth factor receptor 1 (*Fgfr1*), angiogenesis-promoting guanine nucleotide exchange factor for the Rho family of Ras-related GTPases *Vav2*, and a homeobox transcriptional repressor *Msx1* (Table S3), while *Vegfa/b* or *Vegfr1/2/3* were not differentially expressed. Consistently, intratumoral levels of vascular endothelial growth factor (Vegf) did not differ between *Dnmt3a*^*+/Δ*^ and control *Dnmt3a*^*+/+*^ BM chimera (Fig. S2 A). Overall, these results are consistent with our findings of increased proliferation within the epithelial layer of regenerating colons in *Dnmt3a*^*+/Δ*^ chimeric mice likely leading to a more advanced CAC pathology.

### Treatment with an angiogenesis inhibitor eliminates the tumor-promoting effect of *Dnmt3a*^*+/Δ*^ BM

**Figure 4.**
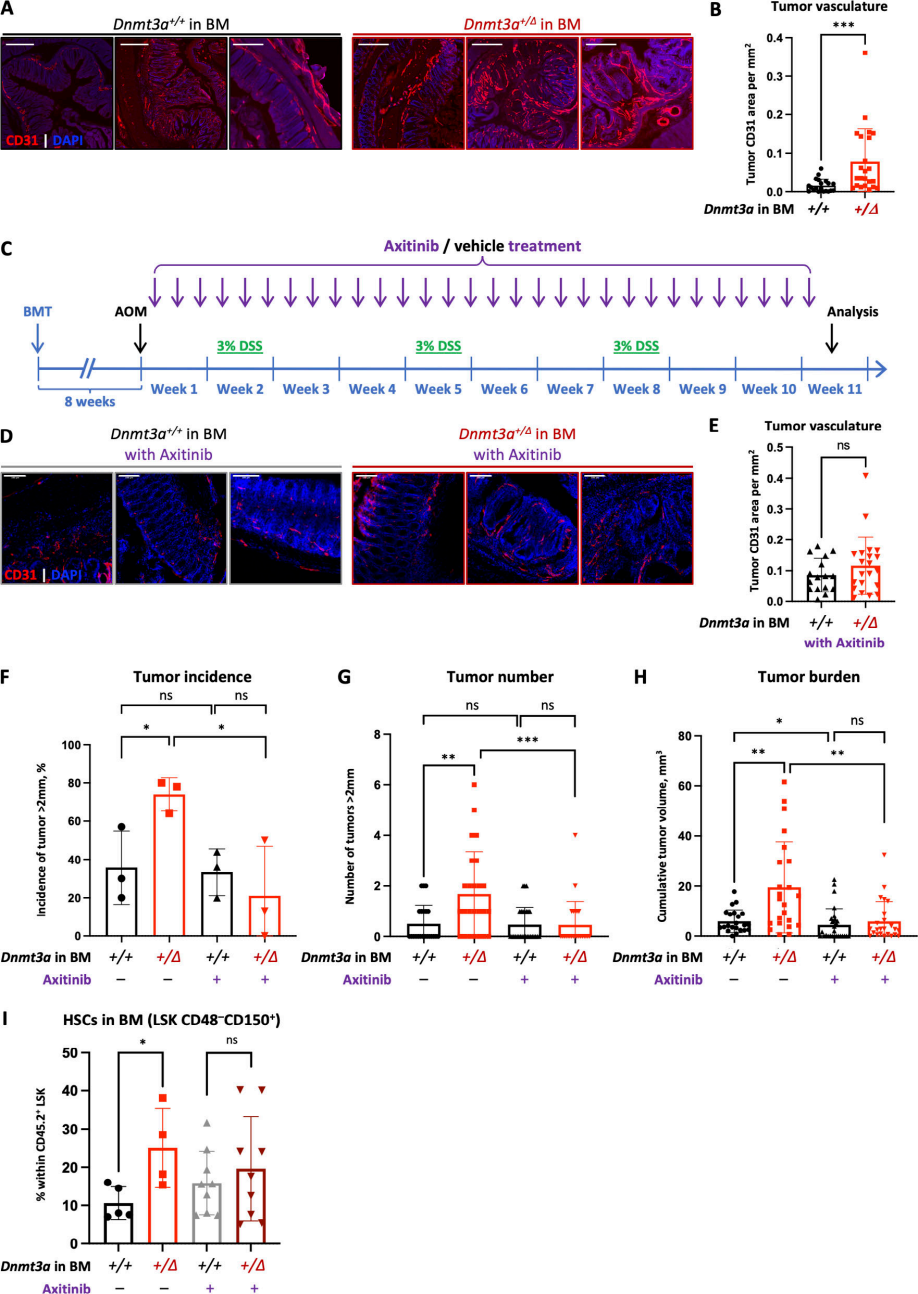
**Targeting increased tumor angiogenesis in *Dnmt3a***^***+/Δ***^**-CH animals mitigates accentuated CAC phenotype. (A and B)** Representative examples of CAC tumors immunofluorescently stained for endothelial marker CD31 (A, red) and quantification of its area within each tumor (B, *n* = 20–23, Mann–Whitney test, P = 0.0003) demonstrate enhanced cancer angiogenesis in colons from *Dnmt3a*^*+/Δ*^ chimeras compared to *Dnmt3a*^*+/+*^-transplanted controls; DAPI was used to visualize nuclei (blue). Bar, 100 μm. **(C–H)** Treatment with small molecule angiogenesis inhibitor axitinib (25 mg/kg per os, three times per week) mitigates elevated CAC tumorigenesis in *Dnmt3a*^*+/Δ*^-CH mice. Experimental timeline of axitinib treatment in CH-CAC animal model (C). Representative images of CD31-stained (red) colon tumors from *Dnmt3a*^*+/Δ*^ chimeras and *Dnmt3a*^*+/+*^-transplanted controls treated with axitinib (D) and quantification of CD31 area within each tumor (E, *n* = 16–21, Welch’s *t* test, P = 0.22) demonstrate that the treatment is effective in suppressing vascular density in both groups. DAPI was used to visualize nuclei (blue). Bar, 100 μm. Proportion of animals with large tumors (>2 mm in diameter) after axitinib or vehicle control treatment in three experiments (F, Welch’s *t* test); number of large tumors (>2 mm in diameter) per animal, with or without axitinib treatment (G, *n* = 22–28, Mann–Whitney test); and cumulative tumor burden per animal (H, *n* = 22–28, Mann-Whitney test). **(I)** Treatment with axitinib normalizes CH-derived hematopoiesis. Recipient mice (CD45.1) competitively transplanted with *Dnmt3a*^*+/Δ*^ and control *Dnmt3a*^*+/+*^ BM (CD45.2) were treated with axitinib (25 mg/kg per os, three weekly doses) while being administered DSS. Composition of the CH-derived (CD45.2) BM stem and progenitor compartment was analyzed after 70 d of treatment (*n* = 4–9 from one experiment, Mann–Whitney test). *, P ≤ 0.05; **, P ≤ 0.01; ***, P ≤ 0.001; ns, not significant.

To further investigate our finding of an enriched angiogenesis-related gene expression signature detected by RNA-seq, we performed immunofluorescent staining of colon tumors using anti-CD31 antibody that marks endothelia. CAC tumors from animals grafted with *Dnmt3a*^*+/Δ*^ BM had increased CD31 staining area compared with *Dnmt3a*^*+/+*^-reconstituted controls, consistent with more dense vascularization (Fig. 4, A and B) known to promote tumor growth. Hence, we hypothesized that cancer angiogenesis in animals with experimental CH may be targetable therapeutically to mitigate accentuated tumor phenotype. Axitinib is an orally bioavailable small-molecule angiogenesis inhibitor that is FDA-approved for treating advanced renal carcinoma with clinical trials for other cancers ongoing (Gross-Goupil et al., 2018). Axitinib is a tyrosine kinase inhibitor of Vegf receptors −1, −2, and −3 that is also active against platelet-derived growth factor receptor (PDGFRβ) and stem cell factor receptor c-KIT/CD117 as part of its potent anti-angiogenic mechanism of action (Escudier and Gore, 2011). After confirming that axitinib potently inhibited cancer angiogenesis in a CAC model (Fig. S2, B and C), we tested if it also abolished the colon tumor-promoting effect of *Dnmt3a*^*+/Δ*^ BM (Fig. 4 C). A 10-wk course of axitinib treatment eliminated the difference in vascular density between genotypes (Fig. 4, D and E). At the same time, a substantially lower proportion of *Dnmt3a*^*+/Δ*^*-*BM grafted animals had large tumors compared with vehicle controls (Fig. 4 F). Treated animals in the *Dnmt3a*^*+/Δ*^*-*BM group developed dramatically fewer large tumors and presented with significantly lower tumor burden per colon (Fig. 4, G and H), while the tumor phenotype in the *Dnmt3a*^*+/+*^-grafted mice was only slightly affected by axitinib administration.

**Figure S3.**
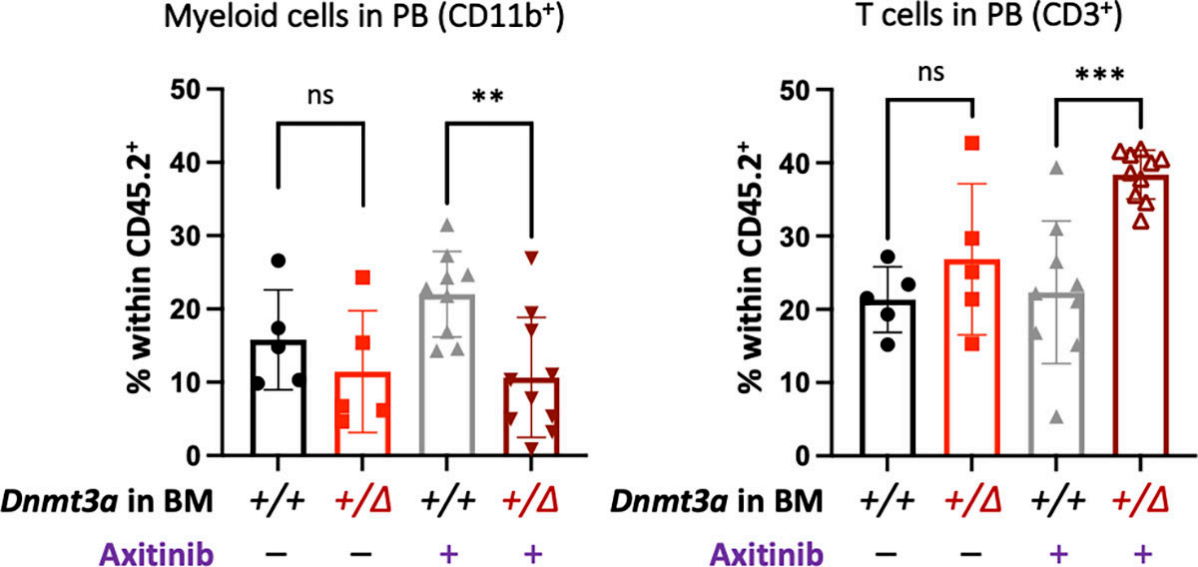
**Treatment with axitinib rebalances CH-derived hematopoiesis.** Recipient mice (CD45.1) competitively transplanted with *Dnmt3a*^*+/Δ*^ and control *Dnmt3a*^*+/+*^ BM (CD45.2) were treated with axitinib (25 mg/kg per os, three weekly doses) while being administered DSS. Composition of the CH-derived (CD45.2) peripheral blood was analyzed after 70 d of treatment (*n* = 5–10 from one experiment, Mann–Whitney test). **, P ≤ 0.01; ***, P ≤ 0.001; ns, not significant.

Since axitinib is a multikinase inhibitor that also binds to the stem cell factor receptor cKit essential to the function of hematopoietic stem and progenitor cells, we wondered if it affected hematopoiesis. While there were no differences in the major lineage composition in the peripheral blood of vehicle-treated mice transplanted with *Dnmt3a*^*+/Δ*^ and control *Dnmt3a*^*+/+*^ kept on DSS, administration of axitinib resulted in a decrease in circulating myeloid cells and a coincident increase in T cells in *Dnmt3a*^*+/Δ*^ BM chimeras (Fig. S3). At the same time, axitinib treatment reversed expansion of the Lin^−^Sca1^+^cKit^+^ CD48^−^CD150^+^ long-term (LT) HSCs in the BM of *Dnmt3a*^*+/Δ*^-transplanted animals (Fig. 4 I). These findings indicate that in addition to inhibiting tumor angiogenesis, axitinib may also act indirectly through rebalancing dysfunctional *Dnmt3a*-CH hematopoiesis in the context of inflammation. Overall, axitinib treatment reversed the heightened CAC tumor phenotype in *Dnmt3a*^*+/Δ*^-chimera, suggesting its potential translational utility for mitigating unfavorable effects of CH in cancer patients.

Although in our study using a CAC model *Dnmt3a*-CH promoted both tumor initiation and progression, consistent with prior pan-cancer retrospective clinical observations (Coombs et al., 2017), this effect is likely context specific. Thus, in patients with metastatic colorectal cancer enrolled in the FIRE-3 clinical trial comparing standard chemotherapy plus cetuximab or bevacizumab, both of which inhibit angiogenesis, presence of CH, and CH-*DNMT3A* specifically, was associated with extended overall survival (Arends et al., 2022). Consistently, in our clinical cohort, patients with CH presented with more advanced disease at cancer diagnosis, although the study was not powered to evaluate survival. These contrasting observations may reflect disparate effects of *DNMT3A* mutations in different branches of the immune system within the tumor microenvironment tipping the balance between anti-tumorigenic and pro-resolving phenotypes (Leoni et al., 2017; Prinzing et al., 2021). Similarly, it is possible that different *DNMT3A* mutation types may exert disparate effects on antitumor immunity. Thus, in our cohort, a patient with *DNMT3A*(R882) CH did not develop distant metastases during the observation period while both patients with non-R882 mutations did. This observation, while anecdotal, lends credence to a possibility of different CH alterations imparting divergent functional consequences. While outside the scope of this study, these specific mechanisms remain to be investigated in the future. Further, the effects of *Dnmt3a*-CH are likely pleiotropic and extend beyond anticancer immunity, illustrated by the identification of changes in tumor metabolism and the striking finding of enhanced tumor angiogenesis in animals with experimental CH. In support of this, targeted inhibition of angiogenesis with an FDA-approved small molecule tyrosine kinase inhibitor axitinib abrogated the CAC tumor-promoting effect of experimental *Dnmt3a*-CH, directly by decreased tumor vascular density and indirectly by normalizing aberrant *Dnmt3a*-CH hematopoiesis. These results identify an actionable therapeutic strategy to mitigate the tumor-promoting effect of coincident *DNMT3A*-CH that can be tested in the clinical setting.

Together, these results highlight a significant difference of molecular pathophysiology effected by genetic alterations within the hematopoietic compartment, despite identical mode of CAC induction. It indicates that alteration of *Dnmt3a* in the BM has a profound impact on molecular pathogenesis of CAC through multiple non-tumor-cell-autonomous mechanisms, some of which may be targetable. These findings, for the first time, solidify the causal relationship between CH and the severity of solid tumors and identify potential therapeutic strategies. Further study is needed to investigate underlying molecular mechanisms including immune involvement.

## Materials and methods

### Patients

Patients with CACs were identified from a database of genomically annotated CAC cases maintained under MSK Institutional Review Board protocols 15-297 and WA0143-14. All participating patients signed informed written consent for matched tumor and normal sequencing (MSK Institutional Review Board protocol 12-245), and next-generation sequencing was performed with the MSK-IMPACT assay (Cheng et al., 2015). MSK-IMPACT is a hybridization capture-based next-generation assay encompassing all exons of >340 genes. It is validated and approved for clinical use by the New York State Department of Health Clinical Laboratory Evaluation Program. The sequencing test utilizes genomic DNA extracted from formalin-fixed paraffin-embedded tumor tissue as well as matched patient blood samples. DNA is sheared and DNA fragments are captured using custom probes. MSK-IMPACT contains most of the commonly reported CH genes with the exception that earlier versions of the panel did not contain PPM1D or SRSF2.

### Mice

Animals were housed at the University of Florida Cancer & Genetics Research Complex specific pathogen–free animal facility; all animal studies were approved by the University of Florida Institutional Animal Care and Use Committee under protocol #201909474. A conditional *Dnmt3a* knock-out line was previously described (Guryanova et al., 2016a; Nguyen et al., 2007). *Dnmt3a*^*+/fl*^ *and Dnmt3a*^*+/+*^ *mice* with *Mx1*-Cre deleter on a C57BL6/J background were generated via in-house breeding. To achieve inducible hematopoietic-specific excision, 8–12-wk-old *Dnmt3a*^*+/fl*^:*Mx1-Cre* and littermate WT control mice received five IP injections of poly(I:C) (#tlrl-pic-5; InvivoGen) every other day. *Mx1*-Cre–driven recombination was validated by PCR using genomic DNA from peripheral blood mononuclear cells 2 wk after the last injection, at which point the BM was harvested and viably banked. To model experimental CH driven by *Dnmt3a* loss of function, BM cells from animals with either heterozygous loss of *Dnmt3a* (*Dnmt3a*^*+/Δ*^) or WT controls (*Dnmt3a*^+/+^) marked by CD45.2 were transplanted into lethally irradiated (10.5 Gy split dose) 6-wk-old congenic WT CD45.1 recipients (strain #002014; The Jackson Laboratory) through tail vein injection. Successful engraftment was confirmed by CD45.1/CD45.2 peripheral blood chimerism 2 mo after BMT. Mice of different genotypes were co-housed to control for possible cage effects. Animals received unique IDs for the purpose of blinding; investigators were unblinded after analyses were complete. Peripheral blood was collected by submandibular puncture. Complete blood counts were obtained using HESKA HT5 automated veterinary hematology analyzer. Both male and female recipients were used with equivalent results.

### CAC induction

CAC is induced in a 10-wk-long protocol. Successfully engrafted mice received a single dose of AOM (Cat# A4586, 10 mg/kg; Sigma-Aldrich) by IP injection. 1 wk later, DSS (AAJ6360622, MW ~40,000; Alfa Aesar) was provided in sterile drinking water at 3% (wt/vol) as the sole source of water during each of the three DSS treatment cycles with 2 wk of sterile drinking water between cycles. As the effect of DSS may vary between mouse strains, genders, and housing facilities, each lot was tested to determine the optimal DSS dosing. Body weights were monitored daily during DSS administration, and animals losing more than 25% body weight were euthanized as past humane endpoints approved by the Institutional Animal Care and Use Committee. Colon microbiota was normalized by mixing cage bedding of all experimental groups every other week to eliminate cage-to-cage variability. Since AOM is a systemic carcinogen, all animals were bled monthly to monitor for hematologic malignancies. Animals with hematopoietic disorders diagnosed by complete blood counts and flow cytometry analysis of peripheral blood mature lineages (Mayle et al., 2013) were excluded as potential confounders. At day 70, animals were bled and euthanized for comprehensive analysis. Flow cytometry data were collected on a 5-laser 16-parameter BD LSR Fortessa instrument and analyzed by FlowJo v10 using the antibody cocktail in Table 2.

**Table 2.** Antibodies used for flow cytometry analysis of peripheral blood mature lineages

Marker	Fluorophore	Per 100 μl	Clone	Manufacturer	Catalog #
CD45.1	PacBlue	0.5	A20	BioLegend	110722
CD45.2	APC	0.3	104	BioLegend	109814
B220 (CD45R)	FITC	0.3	RA3-6B2	BioLegend	103206
CD4	FITC	0.5	GK1.5	BioLegend	100406
CD8	FITC	0.5	53-6.7	BioLegend	100706
CD3	FITC	0.3	17A2	BioLegend	100706
C-KIT (CD117)	PE	0.5	2B8	BioLegend	105808
B220 (CD45R)	APC-Cy7	0.3	RA3-6B2	BioLegend	103224
CD11B	APC-Cy7	0.1	M1/70	BioLegend	101226

For in vivo axitinib treatment studies, axitinib (Cat. # S1005; SelleckChem) was diluted in 0.5% carboxymethylcellulose sodium salt to achieve 2.5 mg/ml suspension. Fully engrafted mice were administered 25 mg/kg axitinib three times/wk (Monday-Wednesday-Friday) by oral gavage throughout the duration of the CAC induction. For the analysis of axitinib’s impact on hematopoiesis, AOM was omitted to avoid its carcinogenic effect as a potential confounder.

### Colonoscopy in live animals

Colonoscopy was performed after second and third DSS cycles using the Tele Pack Vet X mini-endoscopic system (KARL STORZ Veterinary Endoscopy) equipped with a rigid 64301 AA Hopkins Straight Forward Telescope 0° with a 10-cm fiber optic light transmission as previously described (Becker et al., 2006; Lippert et al., 2009; Mottawea et al., 2016; Uronis et al., 2009). Briefly, mice were anesthetized with isoflurane and placed ventral side up on a heating pad. An endoscope was carefully inserted into the rectum up to 4 cm under visual control with slow air flow to keep the colon inflated, and withdrawn slowly while recording the localization and size of colon abnormalities. Colonoscopy videos were independently scored by two blinded investigators using MEICS (Fung and Putoczki, 2018) that combines five scoring criteria (colonic wall thickening [0–3], vascular pattern [0–3], fibrin formation [0–3], stool consistency [0–3], and tumor diameter [0–5], for a total score ranging 0–17).

### Tissue dissection and processing

Colons were dissected immediately after euthanasia. After washing out the feces with 2–3 ml sterile PBS, colons were laid on wet Whatman blotting paper and cut longitudinally. Tumors were counted and dimensions in millimeters were measured with digital calipers used to calculate tumor volume V = (Width^2^ × Length)/2. For histological analysis, each dissected colon was Swiss-rolled from the distal end.

### Immunohistochemistry

Swiss-rolled colons were fixed in 4% paraformaldehyde in sterile PBS for 24 h, transferred to 70% ethanol, and stored at 4°C. Paraffin embedding, sectioning, H&E staining, and Ki67 immunohistochemistry were performed at University of Florida Molecular Pathology Core. Briefly, 4-µm sections were deparaffinized and treated by Trilogy (REF:920P-06; CELL MARQUE) in a 95°C water bath for 25 min. Background Sniper (#BS966M; Biocare Medical) was applied for 15 min to reduce background. Sections were incubated with rat anti-mouse Ki67 (#M7249; 1:50, DAKO) for 60 min, followed by NB Rabbit anti Rat 1:200 for 30 min. The stain was visualized using Mach 2 Rabbit HRP polymer (# RHRP520L; Biocare Medical) and the 3,3'-diaminobenzidine) chromogen (#SK-4105; Vector Laboratories,) with modified Lillie-Mayer CAT hematoxylin counterstain (#CATHE-M; Biocare Medical). Slides were scanned using Keyence BZ-X800 microscope and VHX series software.

### Immunofluorescent staining

Swiss-rolled colons were fixed as described above, placed in a series of 10%−20%−30% sucrose solutions in PBS until sinking, and embedded in OCT compound (Cat# 23730571; Thermo Fisher Scientific). Frozen samples were cut into 12-µm-thick sections at −20°C, bound to positively charged microscope slides (Cat# SM2575; ASI) at room temperature (RT), and stored at −20°C. For immunofluorescent staining, slides were thawed at RT for 20–30 min and tissue permeabilized with 0.2% Triton-X 100 in PBS at RT for 10 min, and then thoroughly washed with PBS. After blocking with 10% donkey serum in PBS for 1 h, slides were incubated with goat anti-mouse CD31 primary antibodies (#AF3628; 1:100; R&D Systems) in blocking buffer overnight at 4°C and stained with secondary AlexaFluor633 donkey anti-goat antibodies (#A21082; 1:200; Invitrogen) for 1 h at RT, three washes with PBS after each incubation, and mounted with Prolong Gold Antifade with 4′,6-diamidino-2-phenylindole (DAPI; P36935; Invitrogen) as a counterstain for nuclei. Images were acquired with a Leica DMi8 confocal microscope equipped with a DFC7000 camera using a 20× HC PL Fluotar objective (Leica) using uniform settings across all slides. The LAS Navigator function was used to generate a merged image of the whole cross-section. All images were processed identically using Fiji/ImageJ (National Institutes of Health).

Tumor vascular density was quantified as CD31 (endothelial marker) staining area per mm^2^ of each tumor using ImageJ v1.53k. First, tumors were manually selected using the DAPI channel of the multiple-layer .lif files of the colon Swiss roll. Next, each tumor was cropped and tumor area (in mm^2^) was measured. To reduce background, uniform adjustments were made to the brightness, contrast, and threshold settings in the CD31 channel for all samples. The area of CD31 staining (in mm^2^) was measured and tumor vascularization was calculated as the ratio of CD31 area to tumor area.

### Measurement of intratumoral Vegf concentrations

Tumors excised from surrounding colon tissue were homogenized in 500 μl of 1% NP-40 lysis buffer (150 mM NaCl, 20 mM Tris HCl, pH 7.5) supplemented with protease and phosphatase inhibitors using 0.5 ml of 800 µm glass homogenization beads in screw-top blast microtubes placed in the Benchmark Bead Blaster 24 Microtube Homogenizer at 6 m/s for 30 s with 2-min breaks between the three cycles. Protein concentration in supernatants was quantified using BCA Protein Assay (Life Technologies). All samples were diluted to a uniform protein concentration of 4 mg/ml and submitted to Eve Technologies Corp. for Murine 31-plex cytokine and chemokine discovery arrays.

### Analysis of the stem/progenitor compartment in the BM

Fully engrafted animals administered with axitinib (or vehicle control) and three cycles of DSS were harvested on day 70. Peripheral blood was collected by a submandibular puncture. BM was isolated from long bones by spin-flush method. Red blood cells were depleted by incubating with ammonium-chloride-potassium lysis buffer (A1049201; Thermo Fisher Scientific) on ice for 15 min. Analysis of the donor-derived (CD45.2^+^) stem and progenitor cell populations was performed on single-cell suspensions by flow cytometry. LT-HSCs were defined as Lineage^−^Sca1^+^c-Kit^+^ (LSK) CD150^+^CD48^−^, granulocyte/macrophage progenitors–LSK CD16/32^+^CD34^+^, using the antibody cocktail in Table 3.

**Table 3.** Antibodies used for the flow cytometry analysis of the BM hematopoietic stem/progenitor compartment

Antigen	Clone	Fluorophore	Manufacturer	Catalog #
CD45.1	A20	PE/CY7	BioLegend	110730
CD45.2	104	AF700	BioLegend	109822
NK1.1	PK136	APC/Cy7	BioLegend	108724
CD11B	M1/70	APC/Cy7	BioLegend	101226
B220	RA3-6B2	APC/Cy7	BioLegend	103224
CD3	17A2	APC/Cy7	BioLegend	100222
GR1	RB6-8C5	APC/Cy7	BioLegend	108424
TER119	TER119	APC/Cy7	BioLegend	116223
CD19	6D5	APC/Cy7	BioLegend	115530
CD4	GK1.5	APC/Cy7	BioLegend	100414
CKIT	2B8	APC	BioLegend	105811
SCA1	D7	Biotin	BioLegend	108104
STREPTAVIDIN		Qdot605	BioLegend	405229
CD34	RAM34	FITC	Invitrogen	2003455
CD16/32	93	PerCP-Cy5.5	BioLegend	101324
CD150	TC15-12F12.2	PE	BioLegend	115904
CD48	HM48-1	PacBlue	BioLegend	103404

### Sample preparation for RNA extraction, sequencing, and analysis

After dissection, each colon tumor was submerged in 200 μl RNAlater (AM7021; Thermo Fisher Scientific) in 1.5-ml Eppendorf tube, flash-frozen in liquid nitrogen, and stored at −80°C. Tumor tissue was thawed on ice and transferred into a blaster tube containing 500 μl buffer RLTplus (1053393; Qiagen) and 200 μl glass beads (12621-148; VWR), then homogenized using BeadBlaster 24 Microtube Homogenizer (Benchmark) at 6 m/s for 30 s three times, with 2-min breaks for cooling. Total cellular RNA was isolated with RNeasy Microprep kit (74004; QIAGEN) and quality controlled on a 4150 TapeStation (Agilent). RNA samples with an RNA integrity number 7.0 or higher were picked for library preparation and sequencing. RNA was subjected to standard Illumina-based RNAseq library preparation and sequenced on an Illumina NovaSeq6000 using a paired-end 100 bp chemistry to an average depth of 49 million reads/sample. Input sequences were trimmed with Trimmomatic; quality control was performed before and after trimming using FastQC. Retained reads (>96%) were aligned to mouse reference transcriptome mm10/build 38 using STAR 2.7.3a; gene and transcript expressions (raw fragments per kilobase of transcript per million mapped fragments) were quantified using RSEM v1.2.31. From a transcript count matrix, differential gene expression was evaluated with DESeq2 using a log2 fold change cutoff of ±1 and a false discovery rate of 5% (Love et al., 2014). GSEA was conducted using the *fgsea* package in R. The volcano plot (Fig. 3 A) and heatmap (Fig. 3 C) were produced using the *EnhancedVolcano* and *pheatmap* R packages, respectively. All R code used to analyze RNA-seq data will be made publicly available at https://github.com/RobinsonTroy/CH_CAC_RNAseq.

### Statistical analysis

Sample size calculation was based on the primary endpoint: tumor burden (mm^3^). The number of animals was chosen to ensure 90% power with 5% alpha to detect a difference between groups of one standard deviation (SD) or larger based on variability and technical drop-out rate observed in pilot experiments. All grouped data are presented as mean ± SD. Statistical significance was determined by unpaired parametric Student’s *t* test and by non-parametric Mann–Whitney test after testing for normal distribution. For samples with significantly different variances, Welch’s correction was applied. Statistical analyses and visualization of the data were performed using Prism 9.0.2 (GraphPad Inc.). For pair-wise comparisons, P values ≤0.05 were considered significant.

### Rigor and reproducibility

For the entire study, both female and male animals were used to control for gender-specific effects. All experimental animals from different genotype groups were co-housed to mitigate potential cage effects. Other variables were kept consistent in all cages and experiments, such as microbiota and DSS quality. Animals developing hematologic malignancies were excluded from analysis as potential confounders. Blinding was achieved by assigning random codes to each animal and sample prior to analysis; investigators were unblinded after results had been recorded.

### Online supplementary material

Supplementary material (available online) includes supplementary figures and tables. Supplementary figures illustrate *Dnmt3a*^*+/Δ*^ and *Dnmt3a*^*+/+*^ BM engraftment and tumor infiltration by CH-derived myeloid cells (Fig. S1), intratumoral Vefg levels and effects of axitinib on tumor vascular density (Fig. S2) and on hematopoiesis (Fig. S3). Tables S1, S2, and S3 summarize RNA-seq data from colon tumors derived from three mice with *Dnmt3a*-CH and four WT-engrafted control animals, including normalized gene counts (Table S1), differentially expressed genes (Table S2), and enriched gene sets (Table S3).

## Supplementary Material

Table S1 shows normalized gene counts in colon tumors from *Dnmt3a*^*+/Δ*^-CH mice (*n* = 4) and *Dnmt3a*^*+/+*^-grafted controls (*n* = 3; RNA-seq).

Table S2 shows differentially expressed genes in colon tumors from *Dnmt3a*^*+/Δ*^-CH mice vs. *Dnmt3a*^*+/+*^-grafted controls (fold change >2, P-adjusted < 0.05).

Table S3 shows HALLMARK pathways (MSigDB) enriched among differentially expressed genes between colon tumors from *Dnmt3a*^*+/Δ*^-CH and *Dnmt3a*^*+/+*^-grafted control mice.

## Data Availability

All data underlying Figs. 1, 2, and 4 are available in the published article and its online supplemental material. The data underlying Fig. 3 and Tables S1, S2, and S3 (RNA-seq) are openly available at the NCBI’s Gene Expression Omnibus under the accession number GSE213178.
